# Optical tomography complements light sheet microscopy for *in toto* imaging of zebrafish development

**DOI:** 10.1242/dev.116970

**Published:** 2015-03-01

**Authors:** Andrea Bassi, Benjamin Schmid, Jan Huisken

**Affiliations:** 1 Max Planck Institute of Molecular Cell Biology and Genetics, Dresden 01307, Germany; 2 Politecnico di Milano, Dipartimento di Fisica, Milano 20133, Italy

**Keywords:** SPIM, Fluorescence, Light sheet microscopy, Optical tomography, Time-lapse imaging, Zebrafish

## Abstract

Fluorescently labeled structures can be spectrally isolated and imaged at high resolution in living embryos by light sheet microscopy. Multimodal imaging techniques are now needed to put these distinct structures back into the context of the surrounding tissue. We found that the bright-field contrast of unstained specimens in a selective plane illumination microscopy (SPIM) setup can be exploited for *in vivo* tomographic reconstructions of the three-dimensional anatomy of zebrafish, without causing phototoxicity. We report multimodal imaging of entire zebrafish embryos over several hours of development, as well as segmentation, tracking and automatic registration of individual organs.

## Abstract (precis)

**Summary:** Bright-field imaging of unstained specimens during selective plane illumination microscopy can provide *in vivo* tomographic reconstruction of zebrafish anatomy.

## INTRODUCTION

Our understanding of embryonic development relies fundamentally on the observation of morphogenesis in living organisms (Keller, 2013; Pantazis and Supatto, 2014). Fluorescence light sheet microscopy, such as selective plane illumination microscopy (SPIM; Huisken et al., 2004), has proven to be a powerful tool to image developmental processes *in vivo* with fast, high-resolution optical sectioning over large volumes (Huisken and Stainier, 2009). However, an intrinsic limitation of all fluorescence microscopy techniques, including SPIM, is their inability to image any structures beyond the labeled tissue. The fluorescent structures therefore appear out of context of the sample's anatomy and features such as the growth or migration of fluorescent tissue through the surrounding tissue may therefore be hard to interpret. The anatomy of fixed samples is commonly visualized by imaging autofluorescence, but this is not an option for *in vivo* studies because detection of the low-level, non-specific autofluorescence signal requires high illumination power, causing serious phototoxicity. It is therefore desirable to find a technique that delivers structural information of the unstained tissue to complement the fluorescence data.

Here we show that, without modifying any component of a typical SPIM setup, we can perform a multimodal acquisition that integrates the high-resolution SPIM fluorescence data with optical tomography, purely based on bright-field contrast. We present *in vivo* data of zebrafish to demonstrate that optical tomography is a valuable tool to observe the whole anatomy of living translucent organisms, complementing and improving the analysis of SPIM data. We demonstrate that our multimodal system offers new possibilities to observe localised processes during zebrafish embryogenesis in a broader *in toto* context over several hours or days.

Optical projection tomography (OPT; Sharpe et al., 2002) had been developed to exploit the bright-field contrast of the sample by acquiring several light transmission images (or projections) from different directions; the 3D structure of the sample is then reconstructed using a back-projection algorithm (Kak and Slaney, 1988). Unfortunately, OPT and SPIM are incompatible, as OPT requires a long depth of field, ideally spanning the entire depth of the specimen, whereas SPIM benefits from a shallow depth of field. To overcome this incompatibility, hybrid OPT-SPIM instruments have recently been developed by reducing the numerical aperture (NA) of the detection unit to achieve the long depth of field needed for OPT (Arranz et al., 2013; Mayer et al., 2014). As a consequence, the overall resolution and contrast in the OPT reconstructions are compromised. Moreover, SPIM is considered an ideal technique to image development *in vivo*, whereas tomographic imaging is commonly believed suitable only for fixed and chemically cleared samples and, indeed, only a few *in vivo* applications have been reported (Colas and Sharpe, 2009; Bassi et al., 2011; McGinty et al., 2011). We decided to look for alternative solutions that would achieve tomographic reconstructions of living specimens in a high-resolution, high-NA SPIM setup without any modifications to the hardware.

## RESULTS AND DISCUSSION

### Implementation of optical tomography in a light sheet microscope

**Fig. 1.**
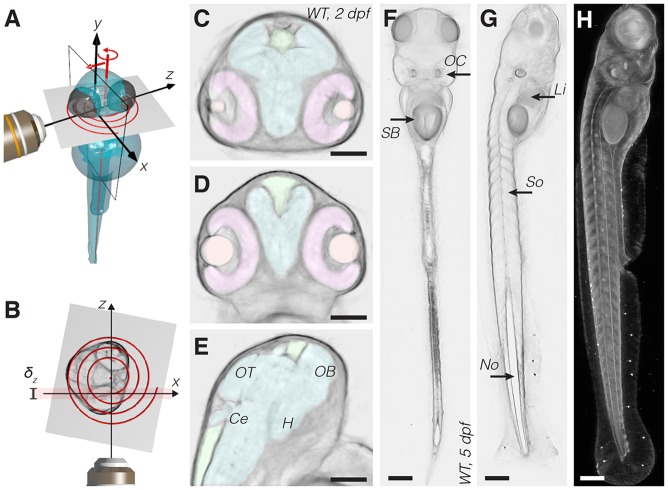
**Optical tomography principles and results.** (A) Scheme of the acquisition system. During the measurement the specimen is translated and rotated through the focal plane of the detection objective lens (*x*,*y*). The specimen is sampled along a spiral. (B) Scheme of the system from the top. The spiral is formed on the transverse section of the specimen. The detection objective's depth of field, δ*_z_*, is highlighted in red. (C-E) Transverse (C), coronal (D) and sagittal (E) slices of a wild-type 2 dpf zebrafish head obtained *in vivo* with optical tomography (reconstructed virtual sections). Segmented head organs: retina (pink), eye lens (orange), brain ventricles (green), brain (cyan). Annotated brain domains: optic tectum (OT), hypothalamus (H), cerebellum (Ce) and olfactory bulb (OB). (F,G) Coronal (F) and sagittal (G) slices of a 5 dpf zebrafish. SB, swim bladder; OC, otic capsule; Li, liver; So, somites; No, notochord. (H) Lateral view of the 3D reconstructed sample. Scale bars: 100 µm.

To integrate an optical tomography approach in a SPIM microscope we took advantage of three features of our existing SPIM setup: (1) fast image acquisition with high frame rate sCMOS cameras; (2) multiview capability, i.e. the sample can be quickly rotated; and (3) LED for back illumination, which can provide transmission images of the specimen. Owing to the relatively high NA of the detection lens (0.3), the depth of field δ*_z_* is only ∼15 µm and does not span the depth of a typical sample in SPIM of ∼0.1-1 mm (Fig. 1). Therefore, a stack of ∼20 transmission images was taken by sliding the sample through the detection objective's plane of focus. The in-focus information was extracted by high-pass filtering the images and a weighted average yielded a projection of the sample with enhanced depth of field (Häusler, 1972) (supplementary material Methods). Since a projection represents an approximation of the line integral of light attenuation along a certain direction, by collecting multiple projections from several directions (typically 360) we created a dataset suitable for tomographic reconstruction; optically sectioned volumes of the samples were obtained by a filtered back-projection algorithm (Kikuchi and Sonobe, 1994; Fauver et al., 2005). No modification to the SPIM hardware or calibration was needed for the tomographic reconstruction. This method can therefore be readily adopted by a large number of systems, including commercial light sheet microscopes and simple SPIM implementations such as OpenSPIM (Pitrone et al., 2013; Gualda et al., 2013).

### Tomographic acquisition is compatible with *in vivo* imaging

The acquisition of 360 image stacks for the tomographic reconstruction was initially performed sequentially, angle by angle, as commonly done for SPIM multiview acquisitions (Swoger et al., 2007). This process was not only time consuming, but also repeatedly exposed the sample to abrupt motion. In order to collect the tomography data more efficiently and avoid any detrimental effects on the sample, we developed a dedicated acquisition process: running the camera continuously at high frame rate, we turned the sample continuously and moved it smoothly through the detection image plane at the same time. Consequently, the image stacks necessary for the reconstruction were acquired in a spiral (Fig. 1A,B; supplementary material Movie 1). Typically, the spiral consisted of 20 complete rotations, images were acquired every 1° at 60 frames per second (fps), and the total acquisition of 7200 images took less than 2 min per specimen. We implemented a real-time processing routine to create projections with enhanced depth of field from the set of images belonging to each angle. Only the final 360 projections were saved to the hard drive for subsequent 3D reconstruction, reducing the amount of data by a factor of 20. The short acquisition times integrate well with the fast SPIM recording and make the measurement compatible with *in vivo* imaging and time-lapse observation of development.

### Optical tomography delivers high-resolution 3D images of unstained zebrafish

To evaluate the quality of optical tomography we imaged living wild-type zebrafish at different stages of development, from 1 to 5 days post fertilization (dpf) and treated with PTU to remove pigmentation. The tomographic reconstruction has inherent isotropic resolution: the sample could be virtually sectioned and visualized along any desired direction (supplementary material Movie 2). Several regions in a 2 dpf zebrafish head were clearly defined by bright-field contrast in transverse, sagittal and coronal sections (Fig. 1C-E). We applied semi-automatic segmentation (supplementary material Methods) based on gradient detection to highlight structures such as brain domains, brain ventricles and retinas (Mueller and Wullimann, 2005) (colored in Fig. 1C-E; supplementary material Fig. S1 and Movie 3). Organs of a known shape could be detected and segmented in an entirely automatic fashion; for example, the spherical eye lens was localized using a sphere recognition algorithm (Schmid et al., 2013). The resulting segmentations are in agreement with data obtained by confocal microscopy in fixed samples using nuclear staining (Ronneberger et al., 2012). Our technique also offered good penetration depth: we were able to reconstruct an entire 5 dpf zebrafish (Fig. 1F-H) by tiling four acquisitions at 10× magnification. At larval stage (3-5 dpf), we identified and segmented internal organs, such as the liver, intestine, swim bladder and notochord (supplementary material Fig. S2). Hence, the tomographic reconstruction is useful on its own to observe zebrafish anatomy *in vivo* and to annotate, segment and quantitatively measure the volume of several organs.

### The bright-field contrast complements the fluorescence signal

**Fig. 2.**
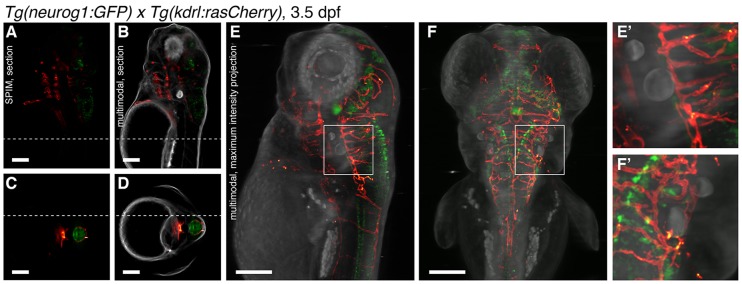
**Multimodal imaging.** (A-D) Sagittal (A,B) or transverse (C,D) slice of a transgenic *Tg(neurog1:GFP)×Tg(kdrl:rasCherry)* zebrafish (3.5 dpf) visualized with (A,C) SPIM or (B,D) SPIM (red/green) combined with optical tomography (gray). The dotted lines in the sagittal sections indicate the position of the transverse section and vice versa. (E-F′) Lateral (E) and dorsal (F) views of the sample created with weighted intensity projection. The boxed regions in E,F are enlarged in E′,F′ to illustrate the fine details in the data. Scale bars: 100 µm.

To assess the value of the multimodal imaging, we recorded and superimposed optical tomography and SPIM volumes of a *Tg(neurog1:GFP)*×*Tg(kdrl:rasCherry)* embryo expressing green fluorescent protein (GFP) in neuronal cells and mCherry in endothelial cells. The fluorescence detected with SPIM was sparse and difficult to assign to any anatomical region (Fig. 2A,C). Only the additional optical tomography information placed the fluorescence data in the proper anatomical context within the zebrafish in all of the reconstructed sections (Fig. 2B,D; supplementary material Fig. S3) and in the entire volume (Fig. 2E,F; supplementary material Movie 4). Registration of the two modalities was straightforward, as both datasets were acquired using the same detector in short succession (supplementary material Fig. S4). Only the relative *z*-position of the reconstructed volume had to be determined by registration (supplementary material Fig. S5). We conclude that the multimodal system provides a comprehensive visualization of the specimen, making it well suited for *in vivo* localization analysis of gene expression in the entire zebrafish.

### Multimodal acquisition of organogenesis in zebrafish

**Fig. 3.**
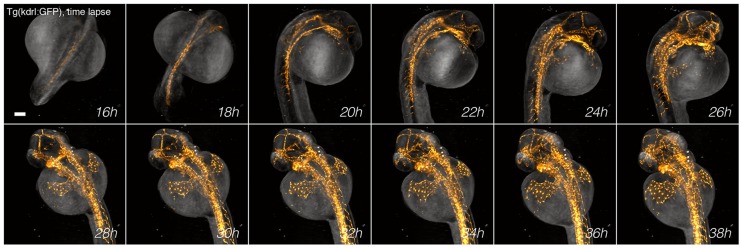
**Long-term time-lapse.** Time-lapse development of a *Tg(kdrl:GFP)* zebrafish from 16-38 hpf acquired every 10 min. SPIM signal (yellow) is superimposed on tomographic reconstruction (gray). The optical tomography data are inverted in all panels. Scale bar: 100 µm.

In order to demonstrate the value of the system for the analysis of embryogenesis, we captured the course of development of a *Tg(kdrl:GFP)* zebrafish from the early embryo to the larval stage at 10 min intervals with both modalities in parallel (Fig. 3; supplementary material Movie 5). The development of the fluorescent vasculature was acquired at single-cell resolution with SPIM, while the tomographic reconstruction was used to visualize not only the outline of the sample but also the entire volume of the zebrafish anatomy to analyze single organ development. An example is shown in supplementary material Fig. S6, where we visualized head development from 36 to 60 h post fertilization (hpf). Since the head undergoes drastic modifications in a few hours of growth, we registered the time-lapse reconstructions onto a reference system based on the fish anatomy; the positions of the eyes were automatically detected at each time point (supplementary material Methods) and used as landmarks (supplementary material Fig. S6 and Movie 6). This procedure allowed us to observe any location of the head during the entire time-lapse together with the formation of cranial vasculature, such as the development of the primordial hindbrain channels over a single section. We conclude that the multimodal acquisition offers the possibility to localize, track and register specific anatomical regions of the sample, facilities that are not readily available using fluorescence modalities.

### Conclusions

We have demonstrated how optical tomography complements light sheet microscopy, providing a 3D reconstruction with label-free bright-field contrast. The additional information is quickly acquired in between the regular SPIM recordings and is practically free of phototoxicity. We introduced an efficient routine based on a spiral acquisition to extend the depth of field of the microscope without sacrificing image resolution and contrast. Only the relevant data were saved for the tomographic reconstruction. The described multimodal acquisition offers a complete picture of the developing sample, which is particularly valuable when the expression of fluorescence reporters is sparse and scattered across different anatomical regions. The ability to create a reference system based on the anatomy of the sample is not only very valuable for time-lapse registration, but also is essential for the analysis of multiple samples: being able to see the outline of the sample, track internal organs and orient the reconstructed data to a common reference system is indispensable in order to compare multiple samples and perform statistical analysis. Abnormal development and morphological defects (such as edemas, damaged yolk), which may be undetectable using fluorescence imaging, are easily detected in transmission and their extent can be quantified during development. Since no staining of the sample is required, the technique is highly suited to the study of emerging model organisms, in which genetic tools and fluorescent transgenic lines are yet to be established.

## MATERIALS AND METHODS

### Fish lines and sample preparation

Zebrafish (*Danio rerio*) adults and embryos were kept at 28.5°C and were handled according to established protocols (Nüsslein-Volhard and Dahm, 2002) and in accordance with EU directive 2011/63/EU as well as the German Animal Welfare Act. Wild-type zebrafish (AB and TL strains) were used for tomographic imaging. The fluorescent transgenic lines *Tg(kdrl:rasCherry)* (Chi et al., 2008), *Tg(kdrl:GFP)* (Jin et al., 2005) and *Tg(fli:GFP)* (Lawson and Weinstein, 2002) were used to image the vasculature with SPIM and *Tg(neurog1:GFP)* (Andersen et al., 2011) to visualize the nervous system. At 24 hpf the embryos were treated with 0.2 mM 1-phenyl 2-thiourea (PTU; Sigma) to inhibit pigmentation. During imaging, the samples were anesthetized with 200 mg/l Tricaine (Sigma) and embedded in low melting point agarose (Sigma) as described previously (Kaufmann et al., 2012): for single time point acquisition, the samples were embedded in 1.5% agarose inside glass capillaries, for long-term time-lapse acquisition the samples were embedded in 0.1% agarose inside fluorinated ethylene propylene (FEP) tubes (S1815-04, Bola). The imaging chamber was filled with E3 medium (Nüsslein-Volhard and Dahm, 2002) containing 200 mg/l Tricaine.

### Setup

We used the SPIM setup previously described by Schmid et al. (2013) (supplementary material Fig. S4). The four-lens SPIM system consisted of four identical water-dipping objectives (UMPLFLN 10×/0.3, Olympus). One of the four objectives was used for detection. Two objectives, perpendicular to the detection objective, were used for double-sided SPIM illumination. The fourth objective was used for transmission illumination. A red LED backlight (MDBL-CR25, MLEK-A080W1LR, Moritex Schott) was placed behind this objective, providing illumination at high NA. For SPIM illumination, a multicolor laser light engine (SOLE-6, Omicron) with a 488 nm (200 mW) and a 561 nm (150 mW) laser was used. The laser beam was split 50/50 and directed through a chopper wheel (MC1F10, Thorlabs) to illuminate the sample consecutively from opposing directions. Each beam was sent onto a resonant galvanometric mirror (1 kHz, EOPC), which pivots the light sheet and reduces shadowing effects in the excitation paths due to absorption in the specimen (Huisken and Stainier, 2007). Light sheets were generated with cylindrical lenses (*f*=50 mm) and projected with telescopes and the illumination objectives onto the focal plane of the detection lens. The focal plane of the detection objective was imaged onto an sCMOS camera (Zyla, Andor). The camera captured images of a field of view of ∼1 mm^2^ at 30-100 fps. A filter wheel (96A354, LUDL Electronics) was used to select the transmission/fluorescence signal at the desired wavelength ranges. The sample was dipped into the medium-filled imaging chamber from the top and was translated along the *z-*axis with a linear stage (M-404.1PD, Physik Instrumente) and rotated with a rotational stage (M-660.55, Physik Instrumente). Although not strictly necessary, it was convenient to position the sample close to the rotation axis before the acquisition (Cheddad et al., 2012). To this end, we designed a sample holder that incorporated a dual axis translation mount (LM1XY, Thorlabs) and allowed the user to manually move the sample in two directions and center it on the rotation axis (supplementary material Fig. S7).

### Spiral acquisition

A LabVIEW (National Instruments) program was implemented to adjust the stage positions, filter wheels and illumination power. During the spiral acquisition of the tomographic dataset the sample was continuously rotated while the translation stage moved the sample along the optical axis of the detection objective (*z*-axis). Typically, 20 complete rotations were performed while the sample was translated over a distance of ∼500 μm. The speed of the translation motor (typically 4 μm/s) and the rotation stage (typically 60°/s) were adjusted according to the desired frame rate (typically 60 Hz). The motors moved continuously from the initial to the final positions while the camera was acquiring the images. To ensure precise synchronization throughout the acquisition, the controller of the motor and the camera received a common digital trigger at the beginning of the acquisition. Typically, 7200 images corresponding to 20 different positions from 360 angles were acquired. The exposure time was typically 16 ms using the rolling shutter mode of the sCMOS camera or shorter (2 ms) using the global shutter mode. We did not observe any differences in the results obtained with the two shuttering modalities. The acquisition of the entire dataset required ∼2 min for tomography. The LED was on during the entire spiral acquisition and its power was manually adjusted before the acquisition to use the full dynamic range of the camera. Acquisition settings, such as frame rate, exposure time and gain, were controlled with a Fiji plugin (Schindelin et al., 2012), which used the Java Native Interface for function calls into the Andor Software Development Kit. The same Fiji plugin was used to read the images from the camera, for real-time processing and to generate and save the projections with enhanced depth of field. For details of depth of field enhancement, see supplementary material Methods and Fig. S8.

### Tomographic reconstruction

The position of the rotational axis in the *x*,*y*-plane was determined as described by Walls et al. (2005) before tomographic reconstruction. Briefly, a series of images was reconstructed with differently assumed *x*-positions of the rotational axis, and the variance of each image was calculated. The reconstructed image with the maximum variance is the least blurred and is closest to the ideal reconstruction. The corresponding position of the rotational axis was assumed to be the correct one.

Reconstructions were performed in MATLAB (MathWorks) using a back-projection algorithm based on inverse Radon transform (Kak and Slaney, 1988). Transverse sections of the sample were created for each *y*-position of the field of view and the reconstruction was repeated slice by slice to create the 3D volumes (supplementary material Movie 2). The reconstruction required 0.1 to 1 s per transverse section (depending on the region of interest) using a single processor of a Xeon CPU (Intel). In order to reduce possible artifacts resulting from non-uniform illumination and the noise of the camera we implemented a background division and a dark noise subtraction routine (Walls et al., 2005) in the acquisition plugin.

For further details of segmentation and visualization, see supplementary material Methods.

### SPIM acquisition

The acquisition of the SPIM stacks was performed at the end of the spiral acquisition. The LED was turned off, the filter wheel on the detection arm was switched to the position corresponding to the desired fluorescence channel and the sample was oriented to the desired angle using the rotational stage. The speed of the translation motor was chosen such that, for the given frame rate (60 Hz), a *z*-spacing between consecutive frames of typically 2 µm was achieved. The sample was moved along the *z*-direction continuously from the initial to the final position while the camera was acquiring the images and the laser was triggered only during each exposure. Typically, 300 frames were acquired in 5 s. One or more fluorescence channels were acquired sequentially and the sample was then rotated to the next detection angle. Typically, four angles were acquired.

### Alignment of SPIM with optical tomography

The back-projection algorithm reconstructed 3D volumes composed of transverse sections centered on the rotational axis of the system. In order to overlap this reconstruction with the SPIM volume, we determined the position of the SPIM stack relative to the rotation axis. Since the two imaging modalities used the same detector, the datasets were inherently aligned along the *x-* and *y*-directions. We determined the *z*-position of the rotational axis using the SPIM data, acquiring stacks of the fluorescent sample from two different views (e.g. θ=0° and θ=90°): one stack was used as a reference, while the second stack was reshaped and registered to the first stack using a 1D translation (supplementary material Fig. S5). The process was performed once per imaging session.

## Supplementary Material

Supplementary Material
