# Supplementary material for: Optical tomography complements light sheet microscopy for in toto imaging of zebrafish development
Source: Development. 2015 Mar 1;142(5):1016–20. doi: 10.1242/dev.116970 (PMC4352980; doi:10.1242/dev.116970)
Supplement: Supplementary Material [file supp_142_5_1016__index.html]

Supplementary Material 

# Optical tomography complements light sheet microscopy for *in toto* imaging of zebrafish development

## DEV116970 Supplementary Material

**Files in this Data Supplement:**

- Supplementary Material
