# Supplementary material for: Optical tomography complements light sheet microscopy for in toto imaging of zebrafish development
Source: Development. 2015 Mar 1;142(5):1016–20. doi: 10.1242/dev.116970 (PMC4352980; doi:10.1242/dev.116970)

Supplementary Methods | Depth of field enhancement, segmentation and visualization.

Depth of field enhancement. Depth of field (DOF) enhancement was achieved by merging a stack of images collected at different z-positions into a single entirely focused projection (Fauver et al., 2005). To create the projection, one could simply sum the stack of images but this would create a blurred projection and consequently a blurred reconstruction, not optimal to visualize the details of the anatomy (**supplementary material Fig. S8**). We therefore employed an algorithm that relies on a high-pass criterion, which was applied to each image in the stack to extract in-focus regions. Given N images I_i, acquired at different z-positions, the projection with extended depth of field, I^E, was created as:

$$I^E = \frac{\sum_{i=1}^{N} I_i \cdot \left|I_i^{HP}\right|}{\sum_{i=1}^{N}\left|I_i^{HP}\right|}$$

where I_i^{HP} is a high pass replica of the acquired image. We used a fast method to generate the high pass images I_i^{HP}, in order to be compatible with real-time processing: we calculated the resulting value from the subtraction of the image I_i with its low pass replica, which was created by applying a moving average filter (with a kernel radius of ca. 10 μm) to I_i.

We tested an alternative way to extend the depth of field, which consisted in the acquisition of a stack of images, deconvolution of the images using the microscope's point spread function, and summing the deconvolved images to create the projection. The deconvolution was performed in Matlab (MathWorks) using a Richardson–Lucy algorithm. The method requires intense computation (not being compatible with real time processing) but has the advantage to quantitatively reconstruct the light attenuation coefficient within the sample. We used the results obtained with deconvolution as a landmark, to assess the quality of the results given by the high pass filter criterion. Visual comparison between the two methods shows good agreement (**supplementary material Fig. S8**).

Segmentation of organs and tracking of the eyes lenses. We developed a Fiji (Schindelin et al., 2012) algorithm to detect the zebrafish eye lenses, taking advantage of their spherical shape. We locally fitted a sphere to each lens using an intensity-based approach. Local search was used to adjust the parameters of a sphere (3D position and radius) to minimize the mutual information between the image data and an artificial sphere, which was constructed by drawing a sphere surface of 5 pixels width onto a black background. For the calculation of the mutual information only pixels inside the sphere were used.

During time-lapse experiments, we registered the reconstructed head volumes using the eye positions as landmarks: the position of the eye lenses was automatically detected in all the reconstructed volumes. From the coordinates of both lenses a rotational matrix was calculated and applied to align the eyes horizontally in the x,y-reference system. The fluorescence volume obtained with SPIM was aligned accordingly, applying the same

rigid transformation. Initial guesses for the eye positions of the first time point were provided manually, for each subsequent time point the results of the previous time point were used for initialization.

Organs and subdomain segmentation. Organ segmentation was performed using the segmentation tools of Amira software (FEI Visualization Sciences Group, USA). The borders of the region were highlighted in sections of the three-dimensional reconstructed volume. This was performed using a feature of the software that forces the highlighted border to lie on an automatically detected edge. The borders were manually highlighted in three to five sections, along three perpendicular directions. The software then allowed the user to automatically create a three-dimensional segmentation volume that included the highlighted borders and interpolated the volume using a gradient-based detection.

Visualization. Sections and 3D volumes were visualized using Fiji (Schindelin et al., 2012). In order to generate weighted intensity projections (**Fig. 2** and **supplementary materials Fig. S3)** of a 3D data stack, each image of the stack was first multiplied by a weighting factor. The factor varied linearly from 1 (first slice) to 0.5 (last slice). Then, a maximum intensity projection was performed. Compared to simple maximum intensity projection this method provided more details on one side of the sample and was useful to visualize the obtained results from different angles.

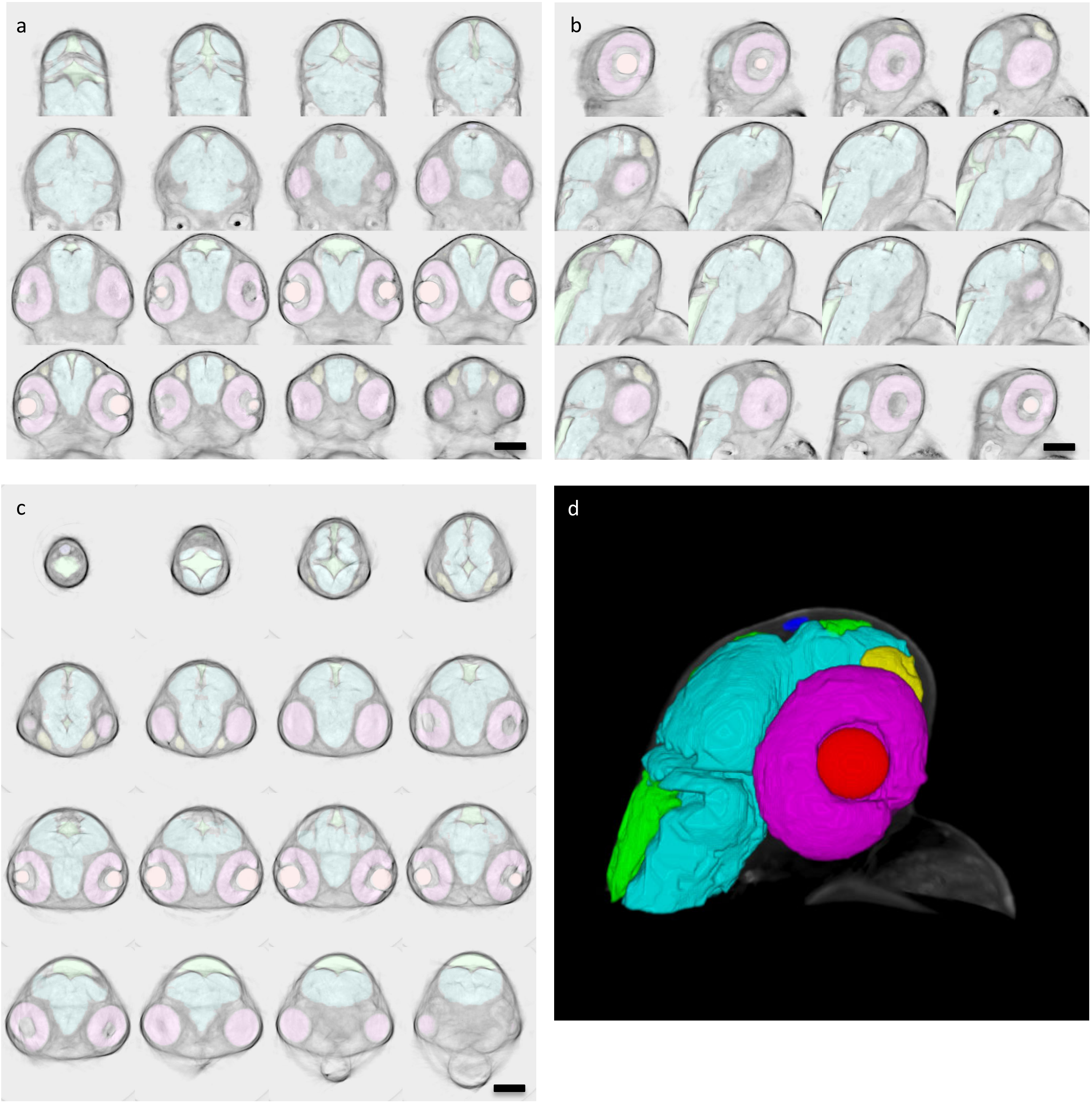

Figure S1 | Sections of a zebrafish head at 2dpf

(a) Coronal, **(b)** sagittal and **(c)** transverse slices of a 2 dpf zebrafish head reconstructed with optical tomography. The superimposed colors indicate segmented head organs: retina (pink), eye lens (orange), brain ventricles (green), brain (cyan), olfactory pit (yellow), epiphysis (blue). **(d)** Three-dimensional reconstruction of the head showing the segmented organs. Slices are spaced 20 µm. Scale bar is 100 µm.

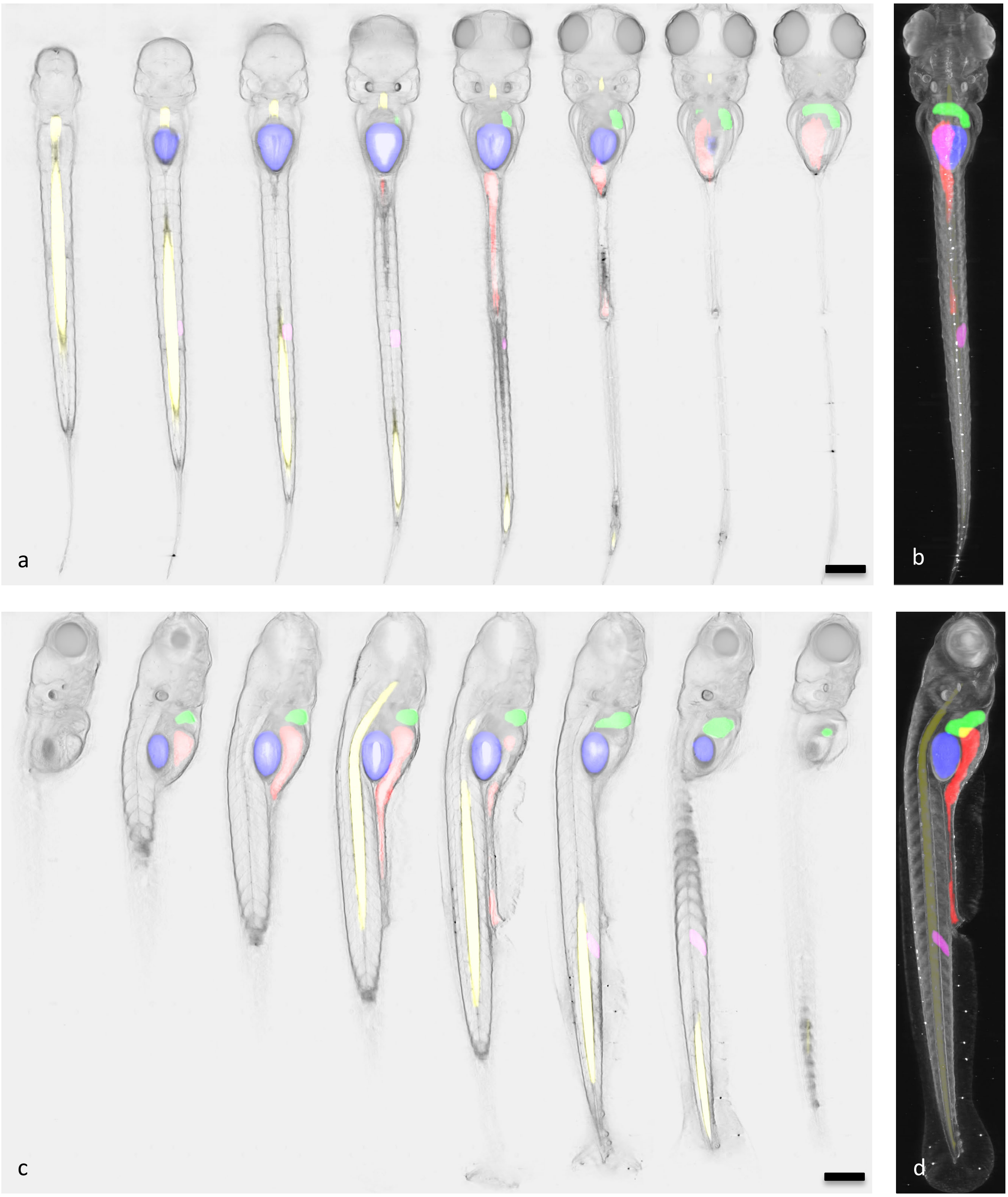

Figure S2 | Sections of an entire zebrafish at 5 dpf

(a) Coronal sections of an entire 5 dpf zebrafish reconstructed with optical tomography. The superimposed colors indicate segmented organs: liver (green), swim bladder (blue), gut (red), notochord (yellow), one single somite (pink). **(b)** Dorsal view of the 3D reconstructed sample. **(c)** Sagittal sections and **(d)** lateral view of the 3D reconstructed sample. Slices are spaced 45 μm. Scale bar is 200 μm.

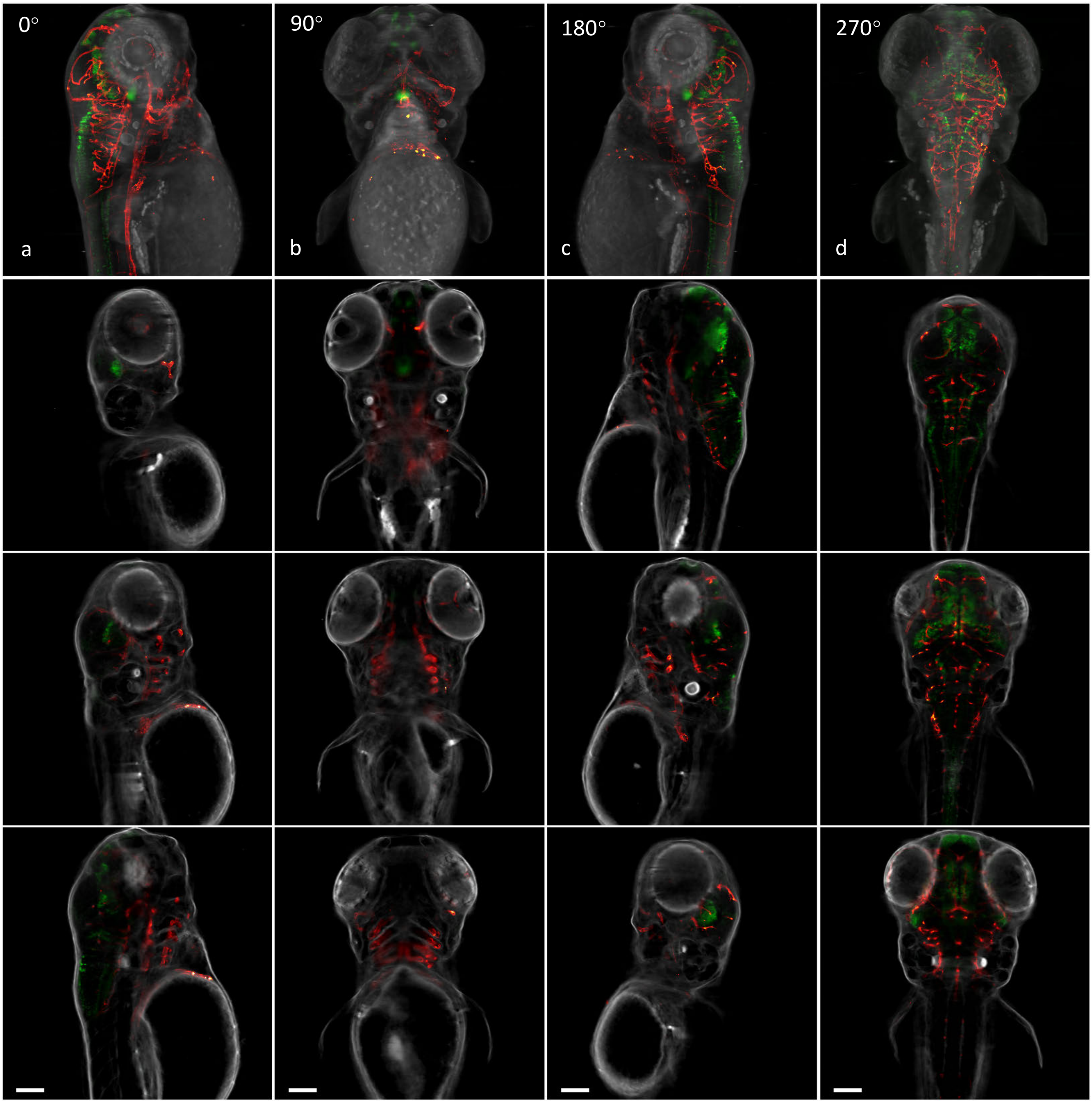

Figure S3 | Multimodal sections

Transgenic *Tg(neurog1:GFP)* x *Tg(kdrl:rasCherry)* zebrafish (3.5 dpf) visualized at different orientations. SPIM (red/green) is superimposed with the inverted optical tomography contrast (gray). **(a)** Lateral view created by weighted maximum intensity projection (first row) and single sections (rest) from the left side to the center of the sample. **(b)** Frontal view, and single sections from the center to the front of the sample. **(c)** Lateral view, and single sections from the center to the right side of the sample. **(d)** Dorsal view, and single sections from the back to the center of the sample. Slices are spaced 65 μm. Scale bar is 100 μm.

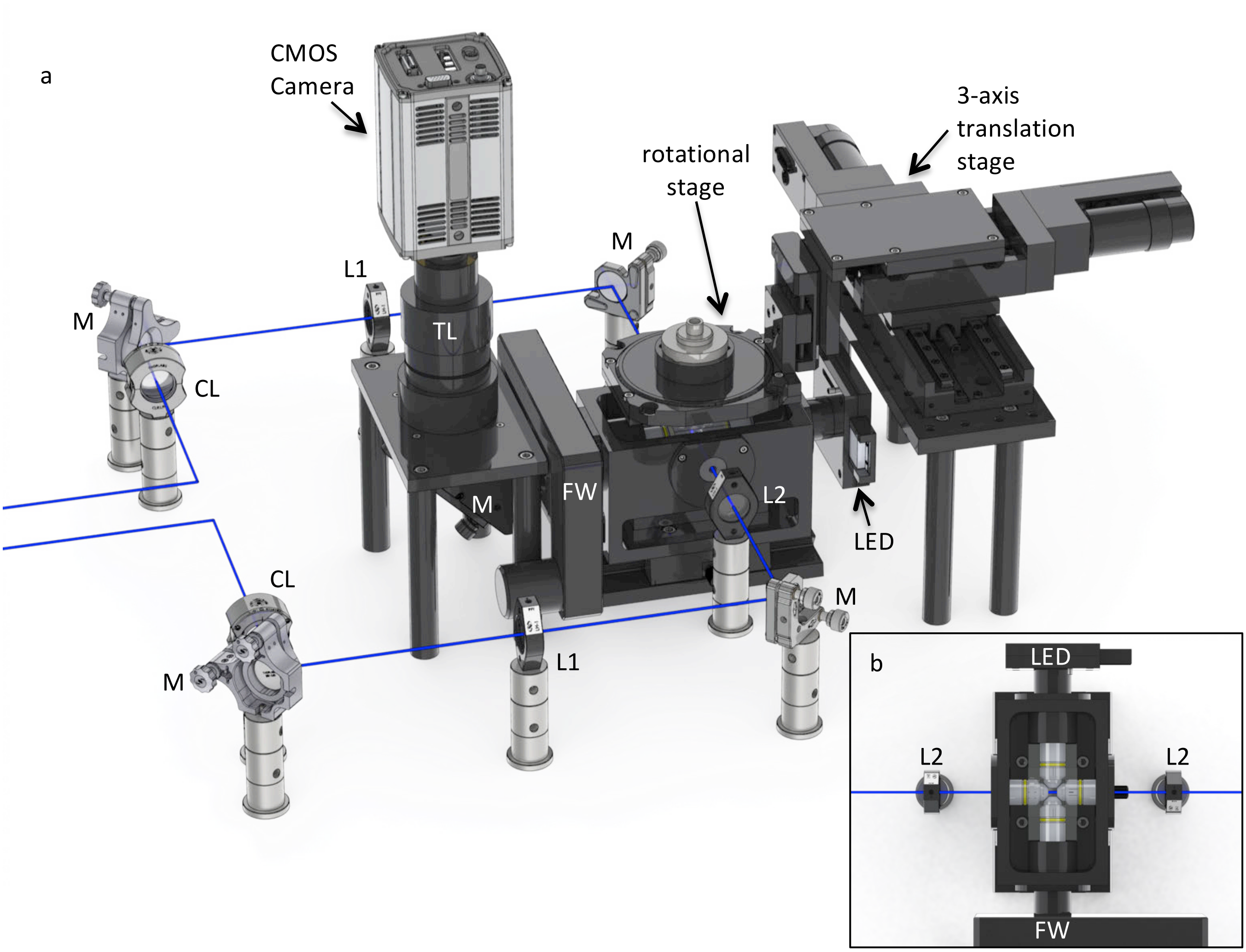

Figure S4 | Optical setup

(a) Schematic of the microscope setup. Each laser beam used for bidirectional SPIM illumination (blue) is passed through a cylindrical lens (CL), a telescope (lenses L1 and L2) and sent to the imaging chamber through an illumination lens. Two 45° mirrors (M) are used to properly align each beam. Brightfield illumination is provided in transmission mode by an LED backlight. The sample dips into the medium-filled imaging chamber from the top. It is held and moved by a fast rotational stage and a 3-axis linear stage. The image of the sample is formed at the detector using a water-dipping objective and a tube lens (TL). The detected light is passed through a filter wheel (FW) and a 45° mirror (M). **(b)** Top view of the imaging chamber. Illumination and detection are provided by four water-dipping objectives. Light-sheets illuminate the sample from two opposite sides (blue beams) and brightfield illumination is provided by an LED backlight, placed at the back of one of the objective. The transmitted or fluorescence light is collected by a fourth objective and passed through the filter wheel (FW).

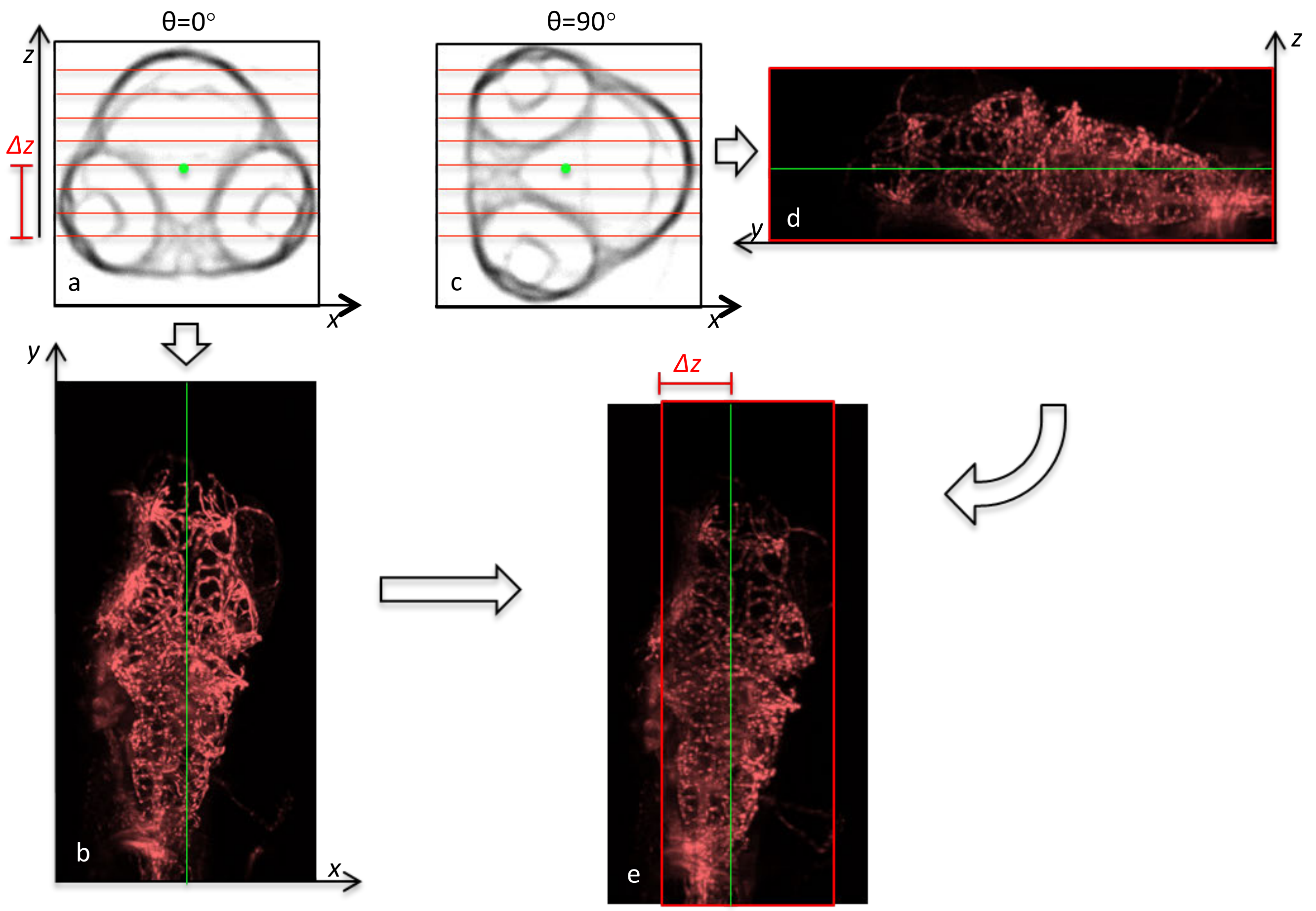

Figure S5 | Alignment of SPIM with optical tomography

(a) Transverse section of the sample oriented at a certain angle θ=0°. Optical tomography based on inverse Radon transform reconstructs squared sections centered on the axis of rotation of the system (green dot). Single planes acquired with SPIM are indicated as red lines in the images. The distance *Δz* (between the first image of the SPIM stack and the axis of rotation) is initially unknown **(b)** Projection along *z* of the SPIM stack acquired with the sample oriented at θ=0°. **(c)** Transverse section of the sample rotated at θ=90°. **(d)** Projection along *x* of the SPIM stack acquired with the sample oriented at θ=90°. **(e)** The distance *Δz* is determined by registering image (d) to image (b), according to the following procedure:

1) Acquire a stack of SPIM images with the sample oriented at angle θ=0°.
2) Sum these SPIM images to create a projection along the direction *z*, as shown in image (b).
3) Rotate the sample to θ=90° and acquire another stack of SPIM images.
4) Reshape this SPIM stack (acquired at point 3) and create a projection along the direction x, as shown in image (d) .
5) Image (b) and image (d) show the sample from the same view. Register these two images translating them horizontally as shown in image (e). The distance between the left side of image (e) and the center of image (d) is the distance *Δz*. This distance indicates the location of the SPIM stack relative to the center of rotation of the sample (green line).
6) Repeat the procedure at four different initial angles in order to determine *Δz* with pixel resolution. Use more angles to achieve sub-pixel resolution.

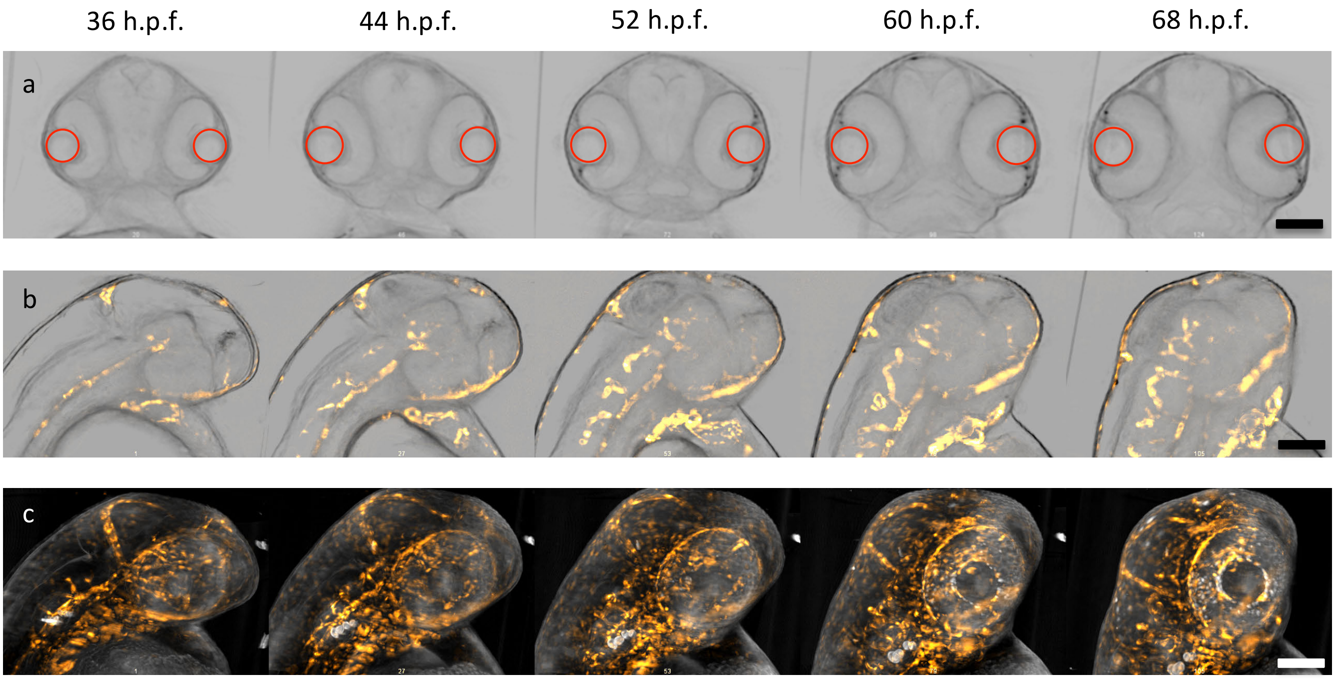

Figure S6 | Registration of time lapse acquisition

(a) Coronal sections of the sample showing the detected spheres (in red), aligned horizontally at every time point of the time lapse. **(b)** Sagittal sections of the sample obtained with optical tomography (gray) superimposed with SPIM acquisition (yellow), showing the formation of the cranial vasculature in a single plane over 1.5 days of development. **(c)** Three-dimensional reconstructions (lateral view) of fluorescence (SPIM) and bright-field contrast (tomography) during time lapse. The sample is a *Tg(fli:GFP)* zebrafish. Scale bar is 100 µm.

All images of the time-lapse reconstructions were registered to a reference system based on the fish's anatomy. The position of the eye lenses was automatically detected in the reconstructed volumes. A reference system with origin in the eyes' center of mass was defined and a rigid transformation was applied to the reconstructed volumes in order to orient the eyes of the zebrafish to the same virtual axis (horizontal) in all the time points of the time lapse (see also Movie 6). The fluorescence volumes obtained with SPIM were aligned accordingly, applying the same rigid transformation.

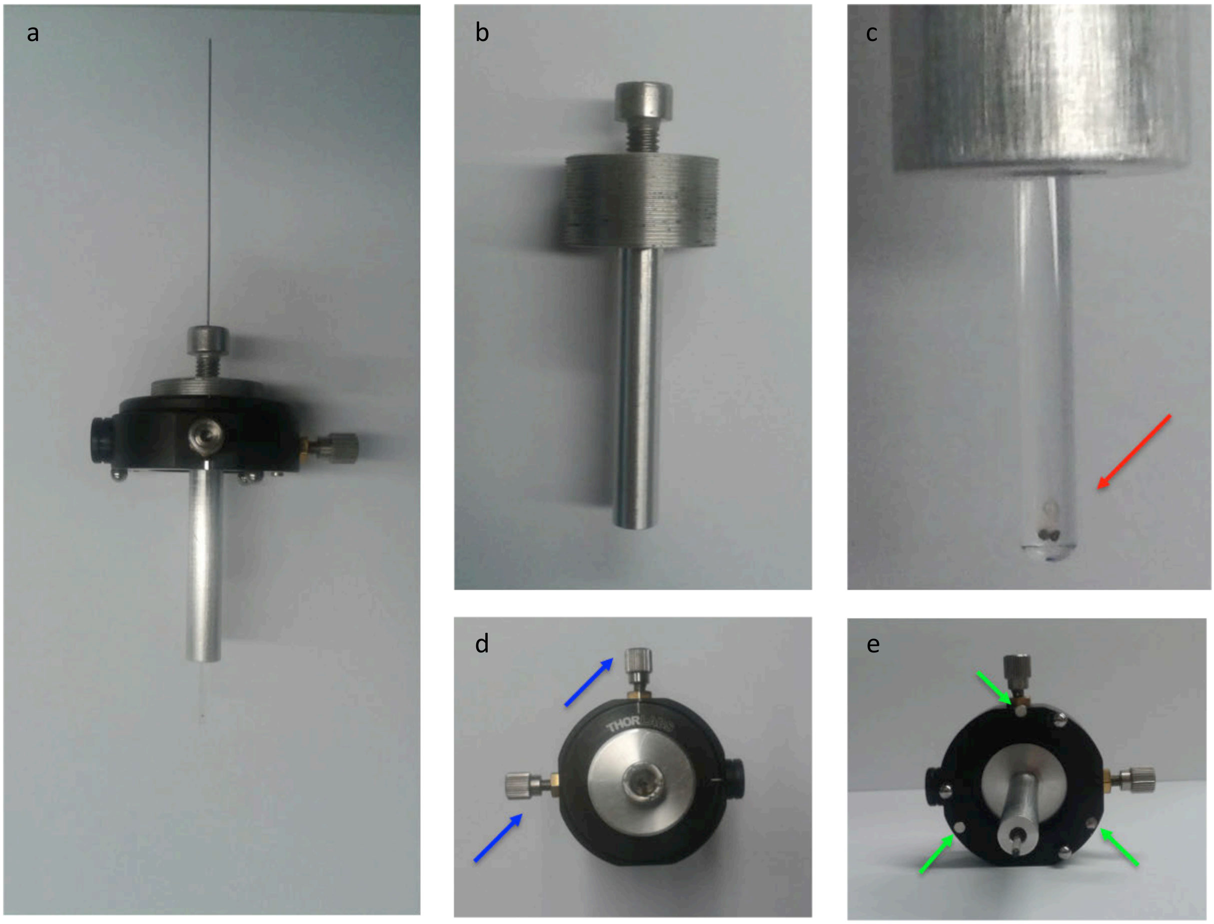

Figure S7 | Sample holder

The sample holder was constructed using a precision xy translation mount (Thorlabs, LM1XY). The two translation screws (blue arrows) were used to move the sample relative to the rotational axis of the rotating stage of the microscope (not shown) and to center the sample in the field of view during the entire 360° rotation. The translation mount was modified to incorporate three magnets to mount the sample holder on the rotating stage (green arrows). The sample (red arrow) is embedded in agarose inside a glass capillary and extruded for imaging.

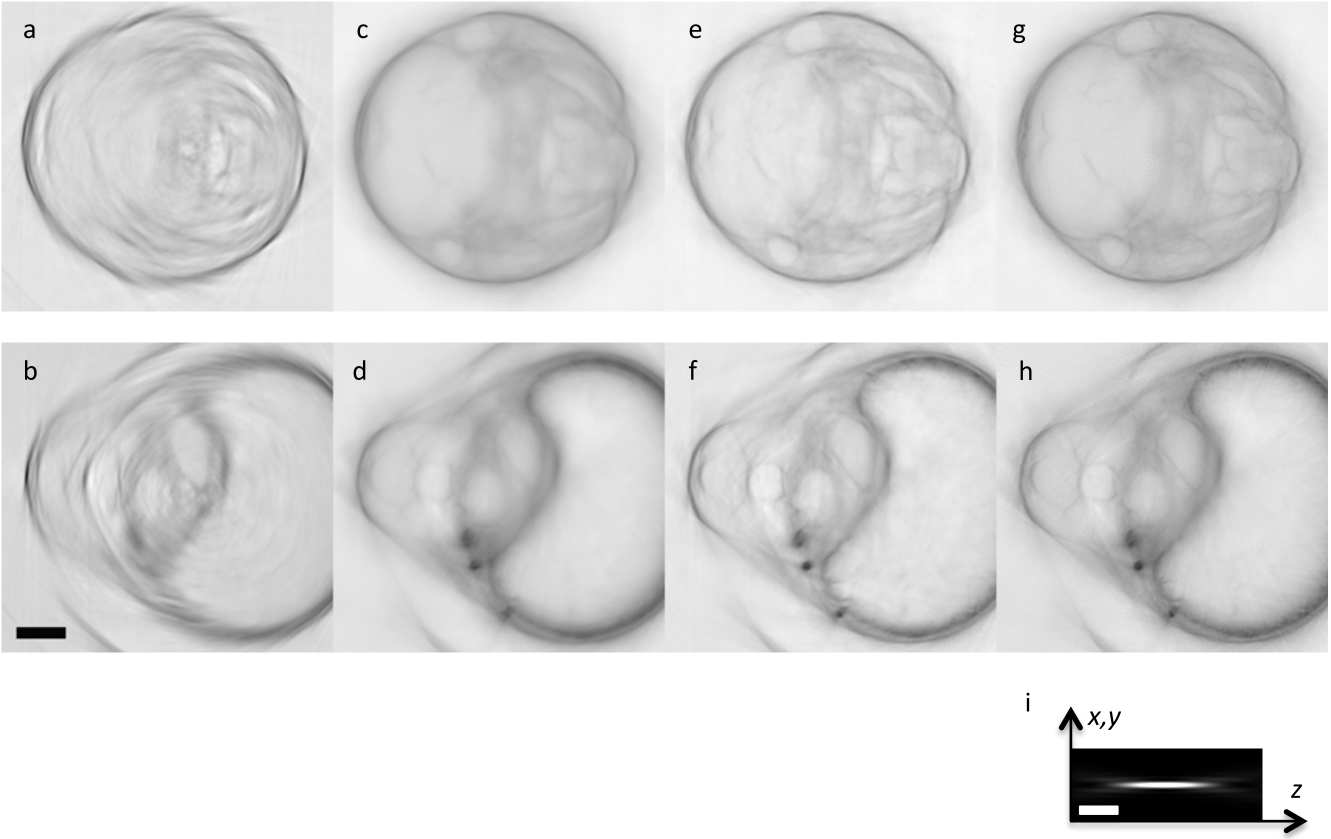

Figure S8 | Comparison of methods for depth of field extension

Reconstruction of 2 transverse sections of a 3 dpf zebrafish at two different locations in the sample: the upper panels show a single section of the head and the lower panels show a section of the trunk. At each of the 360 acquisition angles, a stack of 60 images (spaced 10 µm), was acquired a processed to create the projection. Different methods were used to generate the projections with extended depth of field. **(a,b)** No extension of the depth of field, (**c,d**) average image from full 3D stack,. **(e,f)** high-pass criterion applied to the stack, **(g,h)** deconvolution applied to the stack. Scale bar is 100 µm in (a-h). **(i)** Schematic of the theoretical point spread function of the detection objective. Scale bar is 5 µm in (i).

Movie 1 | Spiral acquisition animation and raw data

During acquisition the sample is continuously translated and rotated across the image plane of the detection objective. The specimen is sampled along a spiral. The raw data acquired during the sample movement are shown on the right hand side.

Movie 2 | Tomographic reconstruction

Sagittal (left side), coronal (center) and transverse (right side) sections through a 4 dpf zebrafish (head and trunk regions) reconstructed with optical tomography. Scale bar is 100 µm.

Movie 3 | Segmented organs

Three-dimensional reconstruction of the segmented organs of a wild-type 2 dpf zebrafish head (also shown in Fig. 1 and Fig. S1). The organs are: retina (pink), eye lens (red), brain ventricles (green), brain (cyan), olfactory pit (yellow), epiphysis (blue). The raw reconstructed data are superimposed to the segmented organs, around half of the sample.

Movie 4 | Multimodal 3D reconstruction

Three dimensional reconstruction of a *Tg(neurog1:GFP)* x *Tg(kdrl:rasCherry)* at 3.5 dpf SPIM contrast (red/green) is superimposed to optical tomography (gray).

Movie 5 | Long term time lapse of development

Development of a *Tg(kdrl:GFP)* zebrafish from 16 h.p.f. to 2 dpf, observed every 10 minutes from 2 opposite views. Fluorescence is shown in yellow, brightfield contrast in gray. Scale bar is 100 µm.

Movie 6 | Registration of time lapse acquisition

Unregistered (left) and registered (right) tomographic reconstructions of a zebrafish during 1.5 days of development (maximum intensity projections). The time-lapse reconstructions were registered to a reference system based on the fish anatomy. The position of the eye lenses (shown in red) was automatically detected in all the reconstructed volumes. A reference system with the origin in the eyes' center of mass was defined (red lines) and a rigid transformation was applied to the reconstructed volumes in order to orient the eyes of the zebrafish over the same virtual axis (horizontal axis).

Movie 1

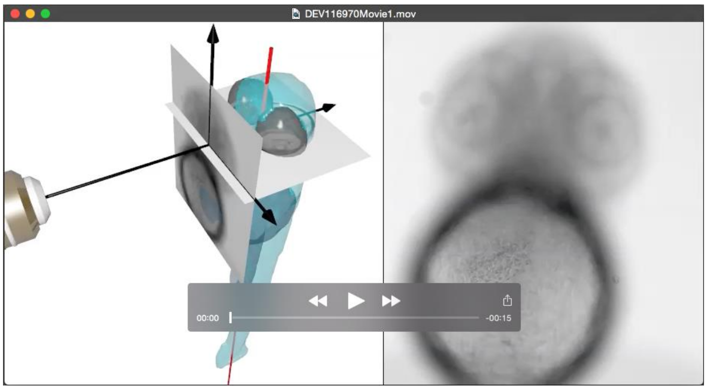

Movie 2

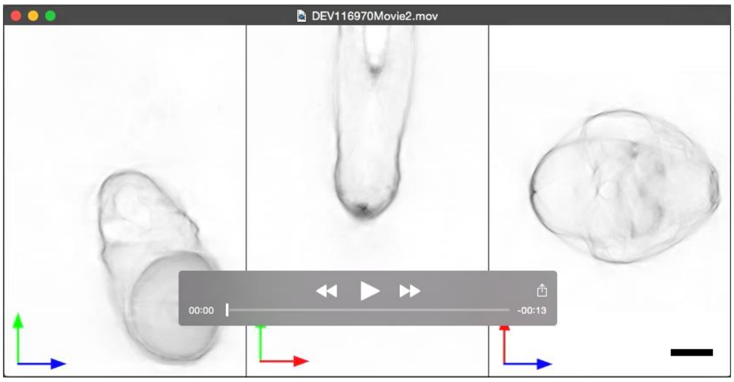

Movie 3

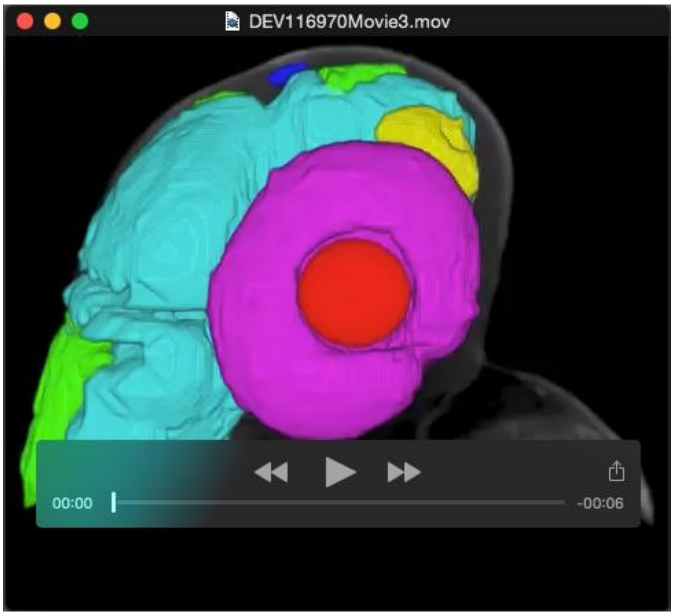

Movie 4

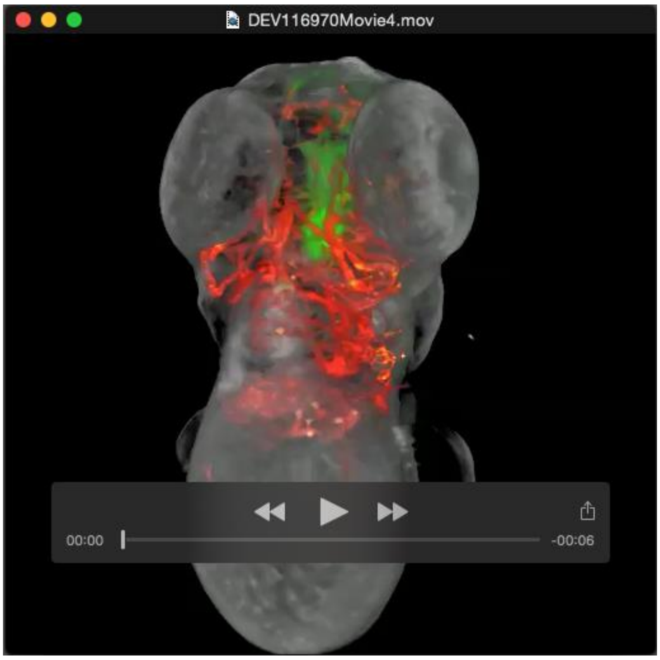

Movie 5

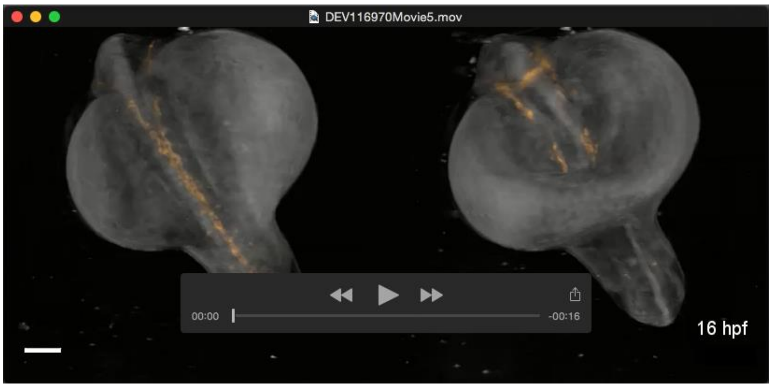

Movie 6

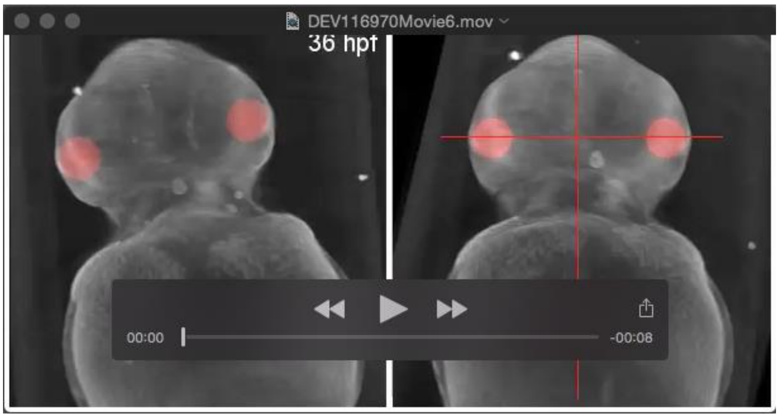

Supplement: Supplementary Material [file supp_dev.116970_DEV116970supp.pdf]
